# Short‐term apparent mutualism drives responses of aquatic prey to increasing productivity

**DOI:** 10.1111/1365-2656.13413

**Published:** 2021-01-11

**Authors:** Fernando Chaguaceda, Kristin Scharnweber, Erik Dalman, Lars J. Tranvik, Peter Eklöv

**Affiliations:** ^1^ Department of Ecology and Genetics; Limnology Uppsala University Uppsala Sweden; ^2^ Present address: Department of Aquatic Sciences and Assessment Swedish University of Agricultural Sciences Box 7050 Uppsala 75007 Sweden

**Keywords:** apparent competition, crucian carp, eutrophication, food web, indirect interactions, mesocosm, resource coupling, top‐down control

## Abstract

1. According to apparent competition theory, sharing a predator should cause indirect interactions among prey that can affect the structure and the dynamics of natural communities.
2. Though shifts in prey dominance and predator resource use along environmental gradients are rather common, empirical evidence on the role of indirect prey–prey interactions through shared predation particularly with increasing productivity, is still scarce.
3. In an 8‐week lake mesocosm experiment, we manipulated both the addition of inorganic nutrients and the presence of generalist fish predators (crucian carp, *Carassius carassius* L.), to test for the effects of indirect interactions through shared predation along a productivity gradient.
4. We found that apparent mutualism (indirect positive interaction) between benthic and pelagic prey strongly affected short‐term responses of aquatic food webs to increasing productivity in the presence of a generalist fish. Increasing productivity favoured the relative abundance of benthic prey, following trends in natural productive lake systems. This led to a shift in fish selectivity from pelagic to benthic prey driven by changes in fish behaviour, which resulted in apparent mutualism due to the lower and delayed top‐down control of pelagic prey at increasing productivity.
5. Our results show empirical evidence that the coupling of multiple production pathways can lead to strong indirect interactions through shared predation, whereby prey dynamics on short time‐scales are highly dependent on the foraging behaviour of generalist predators. This mechanism may play an important role in short‐term responses of food webs across environmental gradients.

## Graphical abstract

In an aquatic mesocosm study, the authors found that switching foraging in a generalist predator can lead to short‐term apparent mutualism between its benthic and pelagic prey. Such indirect prey–prey interactions had important consequences in prey community dynamics and strongly affected the food web response to increasing productivity.

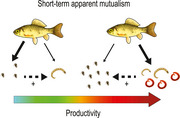

## 1 INTRODUCTION

Food webs are one of the main functional units that regulate ecosystem responses to environmental change. Traditionally, the debate around food web regulation in response to environmental change has focused on whether resources control the abundance of their predators (i.e. bottom‐up control) or predators affect the abundance of resources (i.e. top‐down control). The theories explaining bottom‐up and top‐down control in food webs are originally based on linear food chains (Carpenter et al., 1985; Hairston et al., 1960; Oksanen et al., 1981). However, natural food webs are often dominated by generalist predators that feed on multiple prey items thereby coupling different energy pathways (Polis & Strong, 1996; Rooney et al., 2008; Wolkovich et al., 2014). Under such circumstances, the theory of apparent competition predicts that additional prey will lead to indirect prey–prey interactions that alter the top‐down regulation of the alternative food web pathways (Holt, 1977; Holt & Bonsall, 2017). As environmental gradients often cause changes in prey dominance and predators’ abilities to link different resources (e.g. Bartels et al., 2016; Bartley et al., 2019; Ward et al., 2015), apparent competition theory may help explain and predict changes in food webs along environmental gradients.

Depending on the abilities of predators to control prey populations, indirect prey–prey interactions can range from reciprocal negative effects (–,–), that is, apparent competition, to reciprocal positive effects (+,+), that is, apparent mutualism (Abrams et al., 1998; Holt, 1977; Holt & Bonsall, 2017). In addition, the relative interaction strength of two prey sharing a predator often depends on the time‐scale considered. Over several generations, increasing alternative prey populations are generally expected to strengthen top‐down control on focal prey by subsidizing predator diets thereby increasing predator abundance (Harmon & Andow, 2004; Holt, 1977). However, over short time‐scales, predator foraging behaviour likely outweighs the effects of predator demography as predator life cycles are typically longer than those of their prey (Abrams et al., 1998; Harmon & Andow, 2004; Holt & Kotler, 1987). Sharing a predator would probably lead to short‐term apparent mutualism (Abrams et al., 1998; Holt, 1977), as increasing abundance of alternative prey would release focal prey from predation (e.g. Bety et al., 2002; Nakano et al., 1999; Piovia‐Scott et al., 2019). Yet, differences in predator functional responses to prey abundance may result in multiple alternative top‐down effects (Harmon & Andow, 2004). For instance, with an increasing abundance of alternative prey, satiation in predators reduces per‐capita attack rates and may result in stronger apparent mutualism (Abrams et al., 1998; Holt, 1977; Holt & Bonsall, 2017). Furthermore, according to optimal foraging models (e.g. Pyke, 1984; Pyke et al., 1977), predators may shift from non‐selective opportunistic foraging to fully selective foraging on the highest quality prey, leading to apparent commensalism (0,+) where only the less preferred prey benefits from shared predation. Predators may also adjust selectivity in relation to shifts in prey dominance. For instance, switching predators tend to focus on the most abundant prey, releasing alternative prey from predation (e.g. Holt & Bonsall, 2017). On the contrary, an anti‐switching predator may exert stronger predation pressure on the least abundant prey, if it provides the predator with essential or complementary nutrients that promote fitness (Abrams, 1987, 2010). In such a case, apparent competition (–,–) should occur in the short term, whereby strong prey dominance would tend to indirect amensalistic (0,–) interactions.

To test theoretical predictions of apparent competition, aquatic food webs are particularly good model systems. Aquatic systems may receive substantial amounts of prey subsidies from adjacent terrestrial ecosystems (Bartels et al., 2012), which can affect top‐down control through apparent competition (Nakano et al., 1999; Polis et al., 1997). Furthermore, aquatic food webs are spatially structured in pelagic and benthic pathways, which are coupled by fish that feed on habitat‐specific consumers (Rooney et al., 2006; Vander Zanden & Vadeboncoeur, 2002; Vander Zanden et al., 2011) increasing the susceptibility of benthic–pelagic interactions through shared predation.

The relative importance of benthic and pelagic food web pathways can also substantially shift along environmental gradients (e.g. Bartels et al., 2016; Tunney et al., 2014; Vadeboncoeur et al., 2003), which may further affect indirect interactions through shared predation. For example, Ward et al. (2015) showed that increasing detrital fluxes along the productivity gradient might reduce the abundance of pelagic herbivores through apparent competition (–,–), which in turn had indirect positive effects on pelagic algae biomass through an apparent trophic cascade (Polis & Strong, 1996). Furthermore, pulses of emerging benthic Chironomidae may relax predation on alternative zooplankton prey (Makino et al., 2001). Though theoretical predictions of apparent competition effects in aquatic food webs are well established (Jeppesen et al., 2003; Vander Zanden & Vadeboncoeur, 2002), empirical evidence of apparent competition theory in aquatic systems is still scarce (but see Nakano et al., 1999; Ward et al., 2015).

We tested apparent competition theory in aquatic food webs using an 8‐week mesocosm experiment, where we measured top‐down control of a generalist fish (crucian carp, *Carassius carassius* L.) on its two main prey (benthic Chironomidae and Cladocera zooplankton) along a gradient of nutrient additions ranging from basal mesotrophic to hypereutrophic conditions that were expected to promote the dominance of Chironomidae (Blumenshine et al., 1997). By feeding on benthic Chironomidae and pelagic Cladocera (Penttinen & Holopainen, 1992), crucian carp could functionally link benthic and pelagic pathways through shared predation. In our study, we hypothesize that short‐term apparent mutualism drives responses of benthic and pelagic communities to increasing productivity, whereby increasing relative abundance of Chironomidae along the nutrient gradient will lead to a weakening of top‐down control on alternative Cladocera prey. In addition, we hypothesize that short‐term apparent mutualism is driven by predator foraging behaviour, whereby crucian carp are likely to switch between feeding in benthic and pelagic habitats depending on the abundance of Chironomidae and Cladocera prey, respectively (Begon et al., 2006). Thus, we predict that increasing dominance of Chironomidae will lead to a gradual weakening and a delay of top‐down control on alternative Cladocera prey which is consistent with frequency‐dependent selectivity typical of switching behaviour. In summary, we hypothesize that short‐term apparent mutualism will drastically affect the structure and dynamics of benthic and pelagic prey, with potentially important consequences for the seasonal dynamics of aquatic communities.

## 2 MATERIALS AND METHODS

### 2.1 Experimental set‐up

To investigate changes in top‐down control at increasing productivity levels we used a mesocosm experiment that allowed for controlled environmental conditions in the system while maintaining a complex food web, harbouring pelagic and benthic pathways that are associated with open‐water and mesocosm surfaces respectively. Pelagic pathways are mainly fuelled by phytoplankton and consumed primarily by zooplankton such as Cladocera, whereas benthic pathways are fuelled by periphyton which is consumed by macroinvertebrates such as Chironomidae larvae. Chironomidae larvae can benefit from increased periphyton production and subsidized by settling particulate detritus, which is expected to increase their proportion of community biomass (compared to zooplankton) in highly productive conditions (Blumenshine et al., 1997).

The experiment consisted of 20 white‐opaque, high‐density polyethylene, cylindrical enclosures, 1.5 m deep and 1,000–1,200 L approximately, which were attached to a dock in Lake Erken (59°51′N, 18°36′E) a meso‐eutrophic lake in Central Sweden (Scharnweber et al., 2020). We deployed the mesocosms on 4 July 2017. To each mesocosm, we added a 10 cm layer of sediment from the profundal zone of the lake. Thereafter, we gently filled the mesocosm with lake water filtered through 200 µm mesh, to exclude macro‐zooplankton and fish. To minimize the nutrient fluxes caused by sediment resuspension during water additions, we exchanged the filtered water 1 week after mesocosm filling. Then, we inoculated each mesocosm with zooplankton collected with a 100 µm plankton net (approximately 13 individuals/L) to facilitate even recruitment of large‐bodied zooplankton that are efficient phytoplankton feeders and susceptible to fish predation (Sommer et al., 2012). Insects with aquatic life stages, such as Chironomidae, colonized the mesocosms either from the added sediment or through oviposition from naturally occurring populations in Lake Erken.

### 2.2 Treatments

Detailed information describing the experimental design is available in Scharnweber et al. (2020). In short, we created two equivalent nutrient gradients with 10 mesocosms each, where we added increasing amounts of inorganic phosphorus (P; KH_2_PO_4_) and nitrogen (N; NH_4_NO_3_) following an N: P mass ratio of 11.3:1 (Scharnweber et al., 2020). The target nutrient levels ranged from 20 to 1,000 µg/L total P (TP) and 0.45 to 11.3 mg/L total N (TN) reflecting natural meso‐eutrophic to hyper‐eutrophic conditions. For each gradient, we adjusted nutrient concentrations weekly for the first 2 months of the experiment and biweekly thereafter aiming for 20, 40, 60, 80, 100, 150, 200, 400, 600 and 1,000 µg TP/L. To test the effects of top‐down predation of fish on pelagic and benthic food web pathways, we added two juvenile crucian carp (6.8 ± 1.0 cm; 10.8 ± 0.9 g/mesocosm; *M* ± *SD*) on 25 August 2017 to one of the gradients of 10 mesocosms, using a density within the range of natural populations (Holopainen & Pitkänen, 1985). We reserved the other gradient of 10 mesocosms as fish‐free controls. The experiment ended on 16 October 2017 (8 weeks after fish addition); and over the course of a week, we removed the crucian carp using minnow traps and hand‐netting, and immediately killed them with an overdose of benzocaine. We calculated growth of crucian carp as per cent biomass increase per mesocosm during the 53 days of the experiment.

### 2.3 Sampling

#### 2.3.1 Primary producers and environmental conditions

We estimated phytoplankton biomass, as Chlorophyll *a* (µg/L), and monitored water environmental conditions using the mean of three measurements at the surface, at 1 m depth and at 1.5 m depth from a YSI multiprobe (EXO2 Multiparameter Sonde, YSI Inc.) every 1–2 weeks, rinsing it in lake water before moving it between mesocosms. After fish addition, mesocosm means of physicochemical parameters ranged between 14.6 and 14.9°C, pH 8.5–10.5, conductivity 180–240 µS/cm, 7.8–14 mg O_2_/L, turbidity 1.4–113 FNU.

Every 1–2 weeks throughout the experiment, we also sampled periphyton by scraping the surface of one polypropylene strip (7 cm wide, extending from top to bottom of each mesocosm). We measured periphyton dry weight in pre‐weighed vials after drying for 24 hr at 60°C. Subsequently, we removed inorganic carbon by acidifying a homogenized subsample with 5% HCl, dried it again and then combusted the sample for organic carbon analyses in an elemental analyser (Costech Analytical Technologies Inc.). Finally, we calculated periphyton biomass (g DW m^−2^ week^−1^) by multiplying periphyton dry weight with the carbon content per dry weight of the sample, divided by area and incubation time. Following each periphyton sampling, we scrubbed the periphyton off the walls to decrease its competitive advantage over phytoplankton due to large mesocosm surface area compared to water volume. As the surface area of the walls was approximately three times that of the sediment surface, we used periphyton growth on the walls to estimate the responses of benthic primary producers to the treatments.

#### 2.3.2 Aquatic invertebrates

Zooplankton were sampled every other week by filtering 5 L of mesocosm water obtained from depth‐integrated samples using a 1.5‐m, PVC tube (~3 L). Each zooplankton sample was immediately preserved with Lugol's solution in 100 ml amber glass bottles and later analysed in image analysis software (Image Pro Plus version 7.0 for Windows, Media Cybernetics Inc.) using an inverted microscope (Leica DM IL LED, Leica, Germany) attached to a 12‐bit camera (QI Click F‐CLR‐12, Teledyne QImaging). We counted subsamples until we reached 200 zooplankton individuals. Zooplankton were identified as *Bosmina* sp., *Daphnia* sp., *Ceriodaphnia* sp., *Diaphanosoma* sp., *Polyphemus* sp., *Scapholeberis* sp., Cyclopoida and Calanoida, and later grouped to Cladocera and Copepoda. As Copepoda are underrepresented in the diet of crucian carp due to their anti‐predatory behaviour (Penttinen & Holopainen, 1992), we refrained from using Copepoda to test apparent competition. Copepoda abundance was neither affected by fish nor by nutrient additions (ANCOVA, *p* > 0.05; data not shown).

We assessed Chironomidae abundance by quantifying the emergence of adult stages using cone‐shaped emergence traps (mesh size: 2 mm; diameter: 61 cm) built inspired by LeSage and Harrison (1979) (see Scharnweber et al., 2020 for more information). The traps were sampled carefully with the help of a small vacuum pipe twice a week from 15 August to 19 September 2017 coinciding with the typical late‐summer peak of emerging Chironomidae from Lake Erken (Sandberg, 1969). The low emergence thereafter impeded the assessment of Chironomidae for the last 4 weeks of the experiment. We killed Chironomidae by freezing and sorted them into species and sexes according to their morphological appearance using a stereo microscope. Example specimens of each species were stored in ethanol for species determination.

### 2.4 Analyses

#### 2.4.1 Prey selectivity model

We assessed prey selectivity by crucian carp based on prey depletion trends over the first week after fish addition (Holt & Kotler, 1987; see Supporting Information). In short, we estimated the relative attack rates of Cladocera and Chironomidae prey $\frac{a_{\text{clad}}}{a_{\text{chir}}}$,

$$\frac{a_{\text{clad}}}{a_{\text{chir}}}\;=\;\frac{\log R_{\text{clad}}\left(1\right)\;‐\;\log R_{\text{clad}}\left(0\right)}{\log[S(0)\;‐\;P(1)]\;‐\;\log S(0)},$$

assuming a type II functional response of the predator to each prey type (Holling, 1965), and negligible effects of new recruitment and predation‐independent mortality on the dynamics of prey abundances across treatments. $R_{\text{clad}}\left(t\right)$ is the observed abundance of Cladocera during a given week. We estimated Chironomidae depletion trends based on Chironomidae emergence, where S(0) is the predicted emergence of Chironomidae after the addition of fish (week 0) until the end of the experiment based on fishless mesocosms. P(1) is the estimated number of Chironomidae from S(0) consumed by predators until 1 week after fish addition based on differences in emergence between fish and fishless mesocosm of same nutrient levels. For analysis and visualization of selectivity trends over changes in prey dominance, we transformed $\frac{a_{\text{clad}}}{a_{\text{chir}}}$ into slope degree angles, where 45°‐slopes represent non‐selective opportunistic foragers, slopes < 45° selective predation towards Chironomidae, and slopes >45° selective predation towards Cladocera (Figure S1b). By analysing trends of prey selectivity in response to changes in prey abundance we tested the following alternative foraging behaviours: (a) non‐selective opportunistic behaviour, (b) optimal foraging sensu Pyke et al. (1977) which predicts a shift from opportunistic foraging to fully selective foraging on the preferred prey, (c) switching behaviour that increases selectivity on the most abundant prey and (d) anti‐switching behaviour sensu Abrams (1987) which increases selectivity of the least abundant limiting prey (Supporting Information: Figure S3).

#### 2.4.2 Top‐down control

To measure top‐down control at each step of the nutrient gradient and for each week we calculated log‐response ratio $\log_{10}\left(\frac{N_{f}}{N_{x}}\right)$, where $N_{f}$ is the abundance of prey in the fish treatment and $N_{x}$ is the abundance of prey in the fishless treatments (Shurin et al., 2002). Strong top‐down control would correspond to highly negative values, referring to differences in orders of magnitude between the presence and absence of fish. To assess the temporal aspects of top‐down control, we also calculated the timing of the maximum top‐down control in each mesocosm, defined as the week with the lowest log response ratio.

#### 2.4.3 Food web dynamics

To address treatment differences in temporal responses of primary producers and prey, we conducted a repeated measures ANOVA per food web compartment, using periphyton growth, phytoplankton Chlorophyll *a*, Cladocera abundance or Chironomidae emergence as response variables, while including fish presence/absence as a factor, nutrient levels as a continuous predictor (centred around the mean) and week as a within subject factor. We used Type III sums of squares, testing all possible interactions among factors and explanatory variables to assess whether effects of fish predation depended on nutrient level, week or an interaction between nutrient level and week.

Prior to any analyses, we log‐transformed TP values as well as all response variables, excluding top‐down control, to fulfil the assumptions of parametric tests. Otherwise, we used alternative nonparametric tests. All analyses were performed with R‐studio in R version 4.0.1 (R Core Team, 2020).

The Uppsala Animal Ethic Committee approved the study (permit number 5.8.18‐03672/2017).

## 3 RESULTS

**FIGURE 1:**
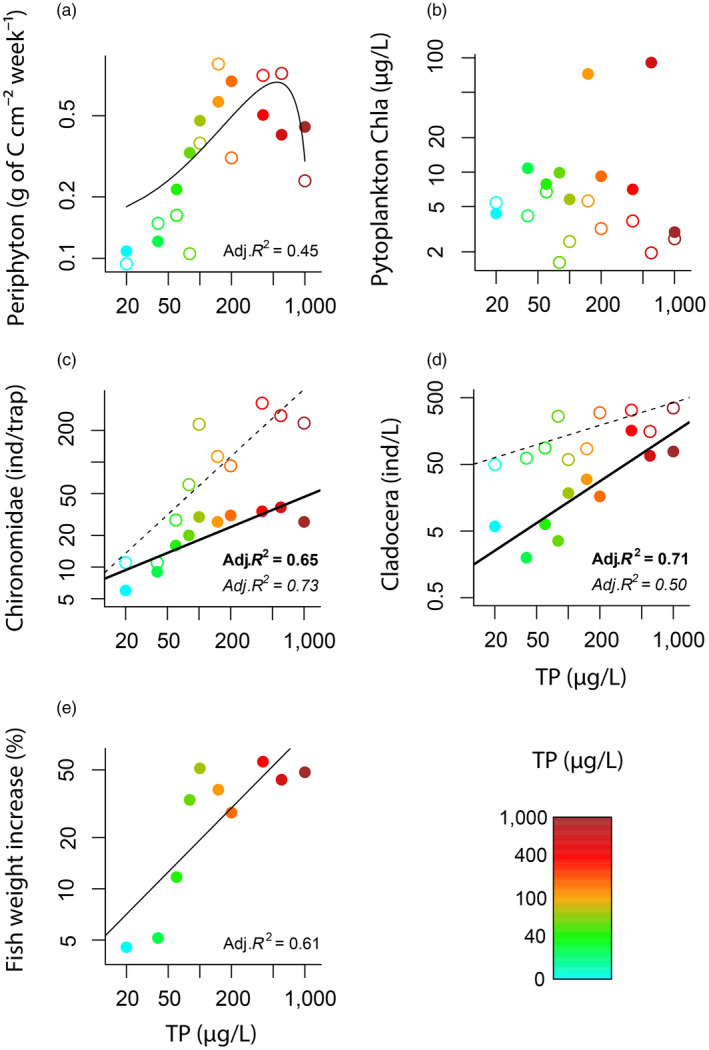
Mean responses of each food web compartment to increasing productivity (depicted as total phosphorus, TP) during the experimental period (a, phytoplankton; b, periphyton; c, Cladocera; d, Chironomidae; e, fish). Filled symbols represent mesocosms with fish, empty symbols represent fishless mesocosms. Units with ind. represent number of individuals. We provide the fit and adjusted *R* ^2^ of significant linear models at *α* = 0.05 for fish mesocosms (bold lines and symbols), fishless mesocosms (dashed lines, italic text font) and in the absence of a fish effect, for both fish and fishless together (solid lines, regular text font). Note that all the axes are shown in logarithmic scale [Correction added on 15 January 2021, after first online publication: Figure 1 corrected to reverse the line types and bold letters]

Increasing nutrients affected the benthic pathway, leading to a fourfold increase of periphyton growth which reached a maximum biomass at around 540 ± 11 µg/L TP (quadratic model estimate ± *SE*; Figure 1a; Table 1a). Phytoplankton biomass did not respond to nutrient additions (Figure 1b; Table 1a). Nutrient additions resulted in a corresponding increase in Chironomidae dominance in the absence of fish, which was depicted by a 40‐fold increase in Chironomidae emergence relative to the sevenfold increase of Cladocera abundance between the lowest and the highest nutrient levels (Figure 1c,d; Table 1a).

**TABLE 1:** Repeated measures analysis for the effects of fish addition (Fish), nutrient gradient (as total phosphorus, TP) and interactive effects between the two on the different compartments of the food web (Phytoplankton chlorophyll *a* (µg/L), periphyton growth (mg of C m^−2^ week^−1^), Cladocera abundance (individuals/L) and Chironomidae emergence (individuals/week). (a) Between‐subject effects of the factors. (b) Within‐subject effects of sampling week and the interactive effects of week with each of the combinations of factors where we applied Greenhouse‐Geisser corrections in the case of deviation from sphericity

(a) Between‐subject effects	Phyto Chl *a*		Periphyton		Cladocera		Chironomidae	
	*F*_(1,16)_	*p*	*F*_(1,16)_	*p*	*F*_(1,16)_	*p*	*F*_(1,16)_	*p*
Fish	**15.67**	**<0.01**	0.46	0.51	**32.94**	**<0.01**	**55.66**	**<0.01**
TP	1.55	0.23	**9.98**	**0.01**	**5.36**	**0.03**	**54.80**	**<0.01**
Fish × TP	0.57	0.46	0.31	0.59	0.36	0.56	**13.07**	**<0.01**

(b) Within‐subject effects	Phyto Chl *a*		Periphyton		Cladocera		Chironomidae	
	*F*_(3.27,62.26)_	*p*	*F*_(1.70,32.38)_	*p*	*F*_(3,48)_	*p*	*F*_(2.09,39.74)_	*p*
Week	**5.01**	**0.02**	**5.23**	**0.02**	**5.77**	**<0.01**	**18.71**	**<0.01**
Week × Fish	3.67	0.05	0.32	0.69	3.89	0.05	**11.39**	**<0.01**
Week × TP	1.64	0.22	3.30	0.06	1.24	0.31	1.27	0.30
Week × Fish × TP	2.36	0.13	0.42	0.63	**3.48**	**0.02**	1.97	0.15

Note

Bold numbers represent significant effects where *p* < 0.05.

**FIGURE 2:**
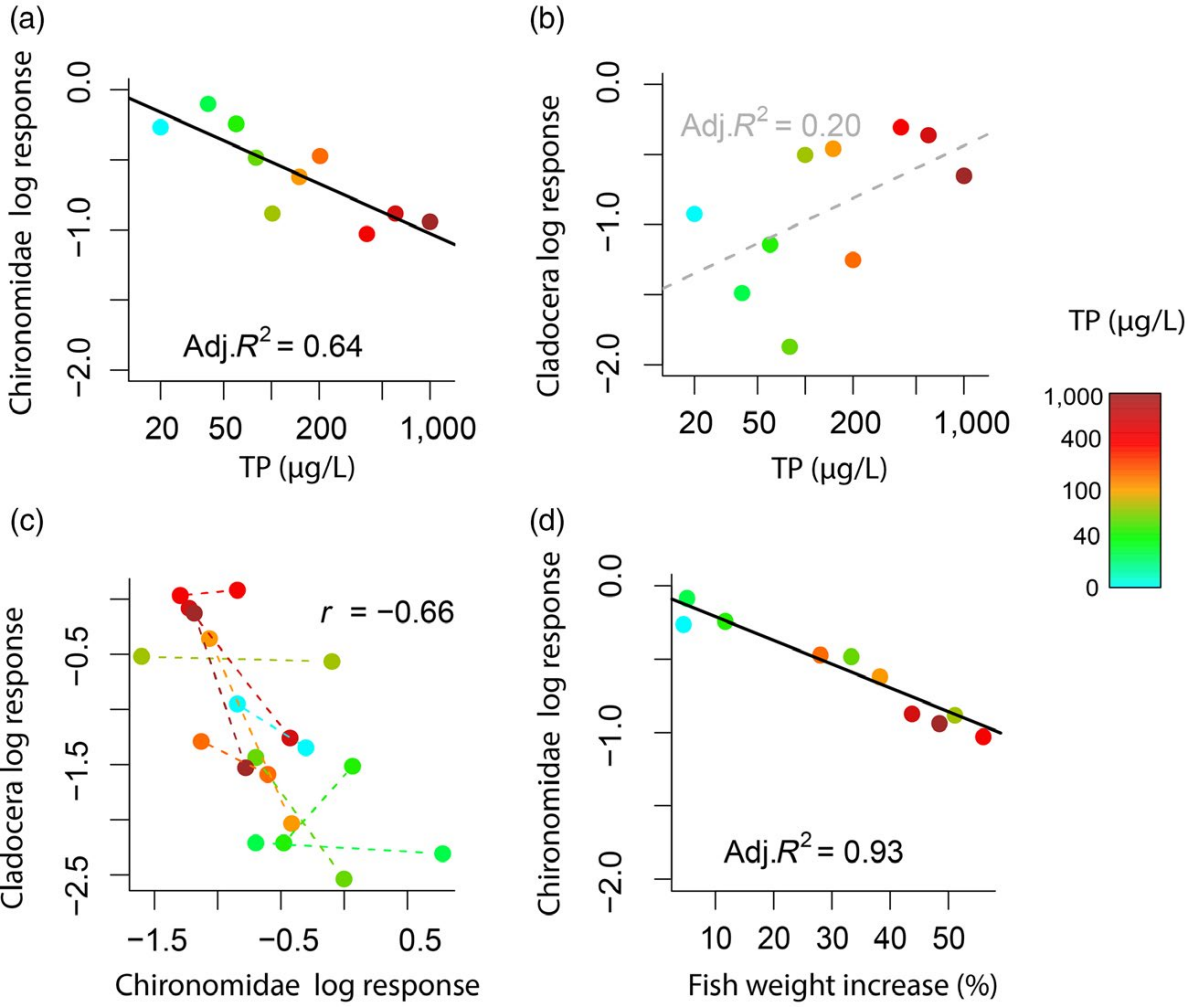
Trends of top‐down control depicted as log response ratios of prey abundance between fish and fishless mesocosms. (a) Top‐down control of Cladocera abundance and (b) Chironomidae emergence (empty squares) at increasing productivity (depicted as total phosphorus TP). (c) Scatterplot of the relationship between top‐down control in Cladocera and Chironomidae (*n* = 20); filled circles joined by dashed lines represent the two samplings after fish addition (week 1 and 3) when both prey were simultaneously monitored in each mesocosm. (d) Top‐down control of Chironomidae emergence in relation to fish weight increase in the mesocosms. A strong top‐down control would correspond to highly negative log response ratios, referring to difference between the presence and absence of fish. We show the fit and adjusted *R* ^2^ of significant linear models at *α* = 0.05 (black solid lines and text font) and non‐significant trends over the productivity gradient (grey dashed lines and text font). In panel c, we show Pearson's correlation coefficient r on the average top‐down control in each mesocosm during the two samplings (*n* = 10)

The relative dominance of benthic and pelagic pathways across nutrient treatments changed together with top‐down regulation, as derived from log responses of prey to the presence of fish. Top‐down control on Chironomidae increased with increasing nutrient additions (Figure 2a; linear regression, *t* = −4.146, *p* = 0.003), weakening the positive effect of increasing productivity on Chironomidae emergence (Table 1a; Figure 1c). These results occurred together with a non‐significant trend of decreasing top‐down control of Cladocera at higher nutrient levels (Figure 2b; linear regression, *t* = −1.829, *p* = 0.104), which had marginally non‐significant effects on Cladocera abundances (Table 1a; Figure 1b). When simultaneously comparing the top‐down control on the two prey groups, the strength of top‐down control on Cladocera was negatively correlated with the top‐down control of Chironomidae (Pearson correlation, *r* = −0.66, *p* = 0.001; Figure 2c), indicating that Cladocera and Chironomidae interacted through short‐term apparent mutualism.

Fish growth increased with increasing productivity (Figure 1e; linear regression, *t* = 3.914, *p* = 0.004) and explained a higher variation of top‐down control on Chironomidae (linear regression, *t* = −11.137, *p* < 0.001, Adjusted *R* ^2^ = 0.93; Figure 2d) and on Cladocera (linear regression, *t* = 2.228, *p* = 0.056, Adjusted *R* ^2^ = 0.30) compared to the nutrient gradient. We tested whether increasing body size promoted an ontogenetic shift from smaller Cladocera to larger Chironomidae prey, by analysing the timing of maximum of top‐down control on Cladocera and Chironomidae. Contrary to our expectations, maximum top‐down control of Chironomidae occurred earlier than that of Cladocera (Wilcoxon test, *V* = 36, *p* = 0.006).

**FIGURE 3:**
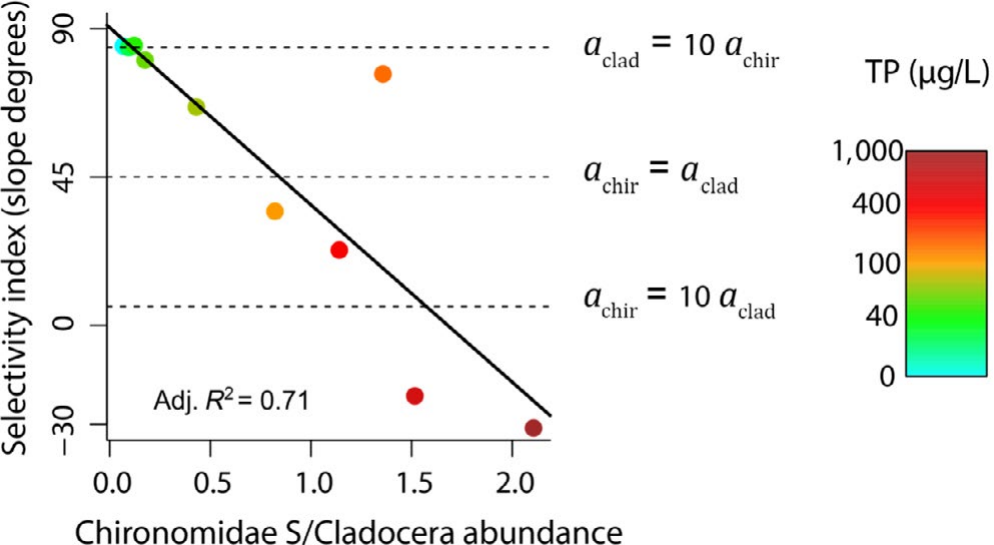
Changes of predator selectivity on Cladocera over the first week after fish additions at increasing relative abundances of Chironomidae (as emerging stock, *S*). Black solid lines and text font represent the fit and adjusted *R* ^2^ for significant linear models at *α* = 0.05. Slopes of 45° depict non‐selective opportunistic foraging; slopes <45° depict selective predation of Chironomidae, whereas slopes >45° depict selective predation of Cladocera. Horizontal dashed lines indicate different levels of prey preference, where $a_{\text{clad}}$ and $a_{\text{chir}}$ represent attack rates towards Cladocera and Chironomidae respectively. In the legend, TP (total phosphorus) depicts differences in productivity across mesocosms

**FIGURE 4:**
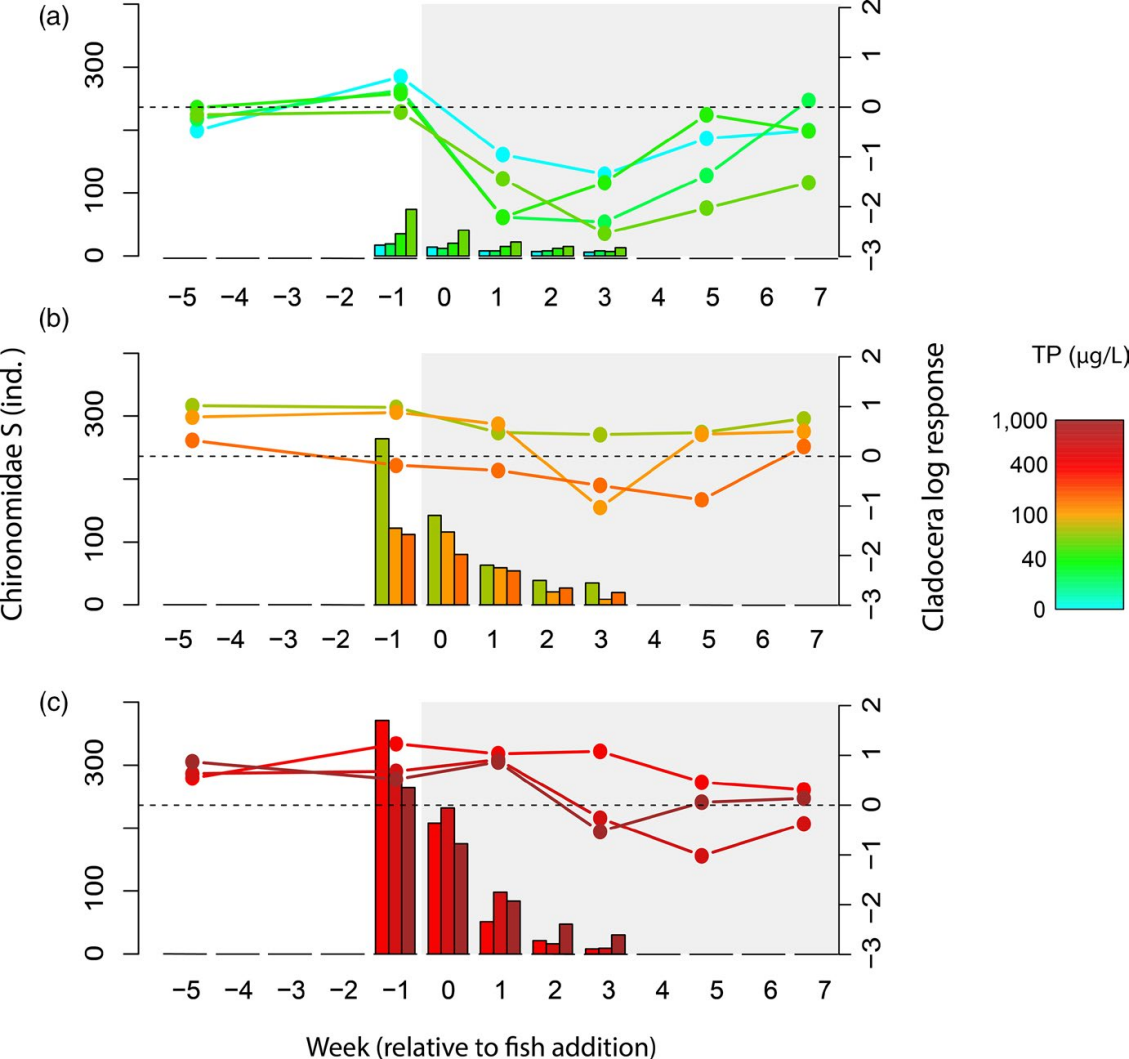
Changes in top‐down control on Cladocera in relation a relative estimate of Chironomidae abundance in the mesocosms. We arranged results based on nutrient levels (as total phosphorus, TP): (a) low (20–80 µg/L TP); (b) medium (100–200 µg/L TP); (c) high (400–1,000 µg/L TP). Bars depict the Chironomidae emerging stock (*S*) per week (left axis), which is an indicator of the abundance of Chironomidae in each fish mesocosms at a given time (Supporting Information). Line‐plots depict the temporal changes in the top‐down control of Cladocera (right axis) in each mesocosm. Horizontal lines represent the threshold under which top‐down control occurs; a strong top‐down control would correspond to highly negative log response ratios, referring to difference between the presence and absence of fish. The shaded part of the graph represents the period in which fish were present in the mesocosms

Instead of coinciding with size‐dependent ontogenetic shifts, the observed apparent mutualism was linked to the type of foraging behaviour of the predator. Crucian carp acted as a switching predator, showing high selectivity for Cladocera at low Chironomidae abundance and gradually increasing selectivity for Chironomidae as they became proportionally more abundant compared to Cladocera (Figure 3; linear regression, *t* = −4.826, *p* = 0.001; Supporting Information). Following predictions of switching behaviour, the top‐down control of Cladocera was delayed when Chironomidae abundance increased at higher productivity (Figure 4), which reflected significant interactive effects of nutrient additions, fish presence and week on Cladocera abundance (Table 1b, Cladocera). The effect of fish predation on Chironomidae emergence depended on week, but the timing of fish predation was not affected by increasing productivity (Table 1b, Chironomidae).

Despite the effects of fish predation on benthic and pelagic primary consumers, only the biomass of pelagic phytoplankton marginally increased with the presence of fish during the experiment (Figure 1a,b; Table 1), suggesting that primary producers were bottom‐up rather than top‐down regulated.

## 4 DISCUSSION

Our results demonstrate that apparent mutualism (+,+) can play a role in structuring benthic and pelagic communities over short time periods, where increasing proportions of benthic prey at increasing productivity led to lower top‐down control of alternative pelagic prey in experimental mesocosms. Indirect interactions through shared predation has long been suggested as an important process in aquatic food webs (Jeppesen et al., 2003; Vander Zanden & Vadeboncoeur, 2002). Yet, mechanistic evidence for the interactions predicted by apparent competition theory has been missing. By comparing marine food webs, Ward et al. (2015) showed that apparent competition (–,–) may drive indirect interactions between aquatic prey, where the increase of benthic detritivores might increase top‐down control on aquatic herbivores by contributing to higher predator numbers. This may not contradict our results, as food webs coupled by generalist predators may first develop short‐term apparent mutualism with no numerical response in the predator (Bety et al., 2002; Nakano et al., 1999; Piovia‐Scott et al., 2019) and then shift to apparent competition in the long run once predator populations show a numerical response to increasing alternative prey (e.g. Bety et al., 2002; Piovia‐Scott et al., 2019; Thomsen et al., 2018) but see Dreyer et al. (2016). Thus, our results emphasize that indirect interactions through shared predation are potentially important drivers of food web responses across environmental gradients, such as increasing nutrient concentrations. However, we encourage studies on longer time‐scales and in larger systems to test the importance of indirect prey–prey interactions, and their type (either mutualism or competition) in a broader ecological context.

As hypothesized, foraging behaviour highly influenced short‐term apparent mutualism. Using an empirical selectivity model based on prey depletion trends, we found that crucian carp acted as a switching predator with respect to the relative abundance of Cladocera or Chironomidae (Supporting Information; Figure 3), which may be expected from a predator that feeds on resources from different habitats (Begon et al., 2006). The switching from Cladocera to more abundant Chironomidae prey at increasing productivity, led to a gradual weakening of the top‐down control of Cladocera that caused the apparent mutualism in our experiment. Such switching behaviour also explained the delay in top‐down control on Cladocera at increasing productivity depicted by the later decrease of Cladocera at high productivity in the presence of fish (Figure 4, Table 1b Cladocera, significant Week*Fish*TP interaction), as predators may have switched later to Cladocera according to their functional response to relative prey abundance (Figure 3). The effect of fish on Chironomidae emergence changed over time, but not in relation to productivity, even though fish controlled Cladocera at lower nutrient levels (Figures 1 and 3). This may have been an artefact of using emergence as a proxy for Chironomidae abundance, as the effects of predation on the abundance of Chironomidae larvae can only be detected after the time they reach the adult stage, which would largely vary depending on species and larval stage of the predated Chironomidae.

Switching behaviour can cause de‐coupled predator–prey dynamics where over‐exploited prey cause other more abundant prey to become disproportionally more vulnerable to the predator, allowing a quicker recovery that dampens prey fluctuations (McCann et al., 2005; Rooney et al., 2006). Therefore, the apparent mutualism and asynchronous predation arising from switching behaviour may lead to shared predation promoting community stability (McCann, 2000; Rooney et al., 2006), which was recently experimentally corroborated in a short‐term pond experiment (Marklund et al., 2019). However, foraging behaviour other than switching may have affected the outcome of shared predation on community dynamics. By consuming prey proportionally to their abundance, opportunistic predators would promote the coupling of predator–prey dynamics, likely reducing the time lags in top‐down control that create higher stability. Optimal foragers (sensu Pyke et al., 1977) would switch from opportunistic to selective behaviour at increasing abundances of preferred prey, shifting from apparent mutualism (+,+) to apparent commensalism (0,+) between prey. Constant transitions between both behaviours should therefore promote lagged predation on less preferred prey, similar to switching predation. Conversely, anti‐switching foraging, which may arise when food is nutritionally complementary or essential (Abrams, 1987, 2010), would make predators prioritize consumption of less abundant, but nutritionally important prey. This would lead to apparent competition (–,–) even at shorter time‐scales, which should destabilize prey dynamics. Overall, predator behaviour, and particularly prey selectivity, may largely influence short‐term community dynamics via indirect interactions through shared predation. Therefore, identifying and compiling behavioural traits and functional responses of generalist predators (e.g. Jeschke et al., 2004), may be important to predict emergent community properties through apparent competition theory.

In addition to foraging behaviour, changes in the body size of predators, may have affected the top‐down control in the mesocosms through the coupling of morphology with performance (Garland Jr. & Losos, 1994). Larger fish are expected to have higher foraging rates to fulfil the metabolic needs of maintaining a higher biomass (e.g. Peters, 1983). Besides, larger crucian carp are known to have lower prey handling times for a given prey size (Paszkowski et al., 1989). Thus, larger fish likely led to a higher consumption prey at higher nutrient additions, which may explain why top‐down control on Chironomidae in the mesocosms was tightly positively correlated to fish growth (Figure 2d). Paszkowski et al. (1989) also showed that optimal prey size increases as crucian carps grow, which may contribute to changes in selectivity from smaller pelagic prey to larger benthic prey through ontogenetic diet shifts (Penttinen & Holopainen, 1992; Werner & Gilliam, 1984). The maximum top‐down control on Chironomidae occurred earlier than for smaller Cladocera prey, suggesting that ontogenetic diet shifts were not the main drivers of top‐down control and apparent mutualism during this experiment. Nevertheless, irreversible changes in predator traits over ontogeny can have strong effects on prey communities (e.g. De Roos & Persson, 2013; De Roos et al., 2003), and thus should be taken into account when studying indirect prey interactions through shared predation.

Many fish are well‐known to rely on both benthic and pelagic resources (Vander Zanden & Vadeboncoeur, 2002; Vander Zanden et al., 2011), yet there is little experimental evidence of the effects of habitat coupling on benthic and pelagic food webs (but see Marklund et al., 2019). By feeding on Chironomidae and Cladocera, crucian carp likely coupled benthic and pelagic habitats in the mesocosms. Therefore, based on our results, we propose apparent mutualism via switching predation as a mechanism by which mobile generalist predators can affect the dynamics of spatially separated food webs. However, the small spatial scale of our experiment may only represent environments where pelagic and benthic pathways are highly connected, such as the littoral zones of lakes (Okun et al., 2005) or during insect emergence, where ascending pupae or nymphs are highly susceptible to predation by pelagic fish (Makino et al., 2001; Wagner et al., 2012). Larger spatial separation of habitats may add an energetic cost for predators switching between habitats and further desynchronize top‐down control on different prey as predators may take a longer time to travel between habitats. Additionally, higher habitat isolation may affect the ability of predators to assess habitat profitability and lead to sub‐optimal habitat switching that would likely result in similar top‐down patterns as opportunistic behaviour. Modelling or experiments at larger spatial scales may help incorporate such potential processes at increasing isolation to upscale the results from mesocosms.

The mechanisms portrayed in this study also suggest that the dominance of a food web pathway may lead to weaker, delayed top‐down control of alternative pathways in the presence of switching generalist predators. Accordingly, generalist predators may respond to changes in prey availability over time by affecting the seasonal succession of alternative prey. In lake ecosystems, fish predation on zooplankton is one of the most important drivers on the succession of plankton communities (Sommer et al., 1986, 2012), hampering the presence and the duration of typical clear‐water phases in lakes by releasing algae from zooplankton grazing. In agreement with a short‐term apparent mutualism hypothesis, Makino et al. (2001) showed that the clear‐water phase can also be related to fish switching prey from water fleas *Daphnia longispina* Mueller to Chironomidae pupae during the peak of Chironomidae emergence. We therefore hypothesize that changes in the dominance and the accessibility of benthic over pelagic prey, due to increasing macrophyte cover (Diehl & Kornijów, 1998), decreasing lake depth (Jeppesen et al., 2003), increasing productivity (Jeppesen et al., 2003; Ward et al., 2015) or due to Chironomidae emergence fluxes or migrations (e.g. Makino et al., 2001; Wagner et al., 2012) may affect the seasonal succession of zooplankton, and its cascading effects on phytoplankton in the presence of generalist predators.

In our study, an increase in Cladocera abundance due to fish switching to feeding on Chironomidae did not have strong cascading effects on the biomass of phytoplankton. This may be due to the monopolization of nutrients by periphyton which likely contributed to the absence of a phytoplankton response to nutrient additions (Figure 1a,b). However, the effects of apparent mutualism on the top‐down control on Chironomidae affected the flux of essential nutrients to terrestrial environments via Chironomidae emergence (Scharnweber et al., 2020). Thus, the effects of indirect prey–prey interactions within aquatic systems can also propagate to adjacent terrestrial ecosystems via cross ecosystem fluxes and affect trophic interactions therein.

Unlike most studies of apparent competition, which directly manipulate prey abundances in the system, our study shows that manipulations of environmental conditions can themselves generate strong changes in prey abundance and prey dominance which enabled us to assess apparent competition. Environmental gradients often result in an asymmetric allocation and transfer of energy in food webs due to idiosyncratic physical, chemical and biological features within different food web pathways (e.g. Bartley et al., 2019; Hayden et al., 2019; Polis & Strong, 1996). In our study, we found higher allocation of nutrients to benthic pathways, leading to an increase in Chironomidae emergence compared to Cladocera abundance in the absence of fish. This could be explained by the high surface to volume ratio of the mesocosms, which may have favoured benthic over pelagic primary productivity (Blumenshine et al., 1997). In natural aquatic systems, rising nutrient levels in the water column tend to promote pelagic over benthic production (Hansson, 1988; Vadeboncoeur et al., 2003), whereas highly eutrophic conditions like the ones in this study tend to promote the dominance of benthic invertebrates subsidized by settling detritus (Jeppesen et al., 2003; Ward et al., 2015).

Irrespective of discrepancies between our mesocosm experiment and natural systems, our results highlight that food web pathways can have asymmetric bottom‐up responses to environmental gradients which can strongly influence top‐down regulation in accordance with apparent competition theory. Over the last 50 years, humans have caused changes in environmental drivers world‐wide (Bartley et al., 2019). Monitoring and understanding asymmetric responses in food webs coupled by generalist predators is therefore important for predicting future changes in food webs in face of environmental change.

## 5 CONCLUSIONS

Our study shows that food web coupling by a generalist predator can strongly affect food web responses to manipulated environmental gradients through strong indirect prey–prey interactions in agreement with apparent competition theory (Holt, 1977; Holt & Bonsall, 2017). This is congruent with recent evidence of generalist predators mediating food web responses across various gradients (Bartley et al., 2019; Ward et al., 2015). Generalist predators are widespread in nature (Polis & Strong, 1996; Rooney et al., 2008), although often underrepresented in empirical food web studies. In addition, many studies focus on single food chains, assuming that influences from other food web pathways are negligible. Our results emphasize that addressing alternative food web pathways under shared predation is important for understanding key food web processes that otherwise might be neglected. We therefore advocate for studying food webs as units that are functionally dependent on generalist predators.

## AUTHORS' CONTRIBUTIONS

F.C., K.S., L.J.T., P.E. conceived the ideas and designed methodology; E.D., F.C., K.S. collected the data; F.C., K.S. carried out data analysis; F.C. led the writing of the manuscript, and all authors contributed substantially to revisions.

## Supporting information

Supplementary Material

## Data Availability

Data are publicly available on DiVa http://urn.kb.se/resolve?urn=urn:nbn:se:uu:diva‐428326 (Chaguaceda et al., 2020).
