# Supplementary material for: Short‐term apparent mutualism drives responses of aquatic prey to increasing productivity
Source: J Anim Ecol. 2021 Jan 11;90(4):834–45. doi: 10.1111/1365-2656.13413 (PMC8048462; doi:10.1111/1365-2656.13413)
Supplement: Supplementary file 1 — Supplementary Material [file JANE-90-834-s001.docx]

# Supporting information. Empirical model to assess the type of predator behaviour based on prey selectivity

**Title:** Short-term apparent mutualism drives responses of aquatic prey to increasing productivity

**Authors:** Fernando Chaguaceda, Kristin Scharnweber, Erik Dalman, Lars J. Tranvik, Peter Eklöv

## Rationale

Our aim was to assess effects of predator behaviour on prey selectivity. We based our analyses on Holt and Kotler (1987) who studied prey selectivity within patches comparing the depletion rates of different prey using the disc equation (Holling, 1965), with a type II functional response of the predator (equation S1):

$\frac{dR_{i}}{dt}= \frac{-a_{i}R_{i}P\left( t \right)}{1+\sum_{j=1}^{2} a_{j}h_{j}R_{j}},$ eqn. S1

where $\frac{dR_{i}}{dt}$is the depletion rate of prey $i$, $a_{j}$ is the instantaneous consumption rate of prey $j$; $h_{j}$, the per-item handling time; and $R_{j}$, the density of prey $j$. Assuming that patch depletion is short enough so that new prey recruitment and predation-independent mortality can be ignored, one can assess prey abundance at time t as (equation S2):

${R_{2}\left( t \right)=R_{2}\left( 0 \right)\left[ {R_{1}\left( t \right)}/{R_{1}\left( 0 \right)} \right]}^{{a_{2}}/{a_{1}}},$ eqn S2

where 0 is the initial time and t is any time point after. Graphically, this model leads to different prey-to-prey abundance trajectories based on prey selectivity (Fig. S1a). Opportunistic, non-selective predation results in a linear trajectory that crosses the origin, whereas fully selective predation toward one prey would lead to a depletion trajectory that parallels the axis of the selected prey (Fig. S1a).

We observed prey-to-prey depletion trends in each mesocosm just after the addition of the predator in order to infer prey selectivity by the predator, where selectivity on prey 1 is a value dependent on ${a_{2}}/{a_{1}}$.

Based on equation S2, intermediate selectivity patterns would lead to logarithmic or exponential curved trajectories (Fig. S1a). To avoid this problem, we applied logarithms in equation S2, and solved the equation such that (equations S2 & S3):

$\frac{a_{2}}{a_{1}}=\frac{\log R_{2}\left( t \right)-\log R_{2}\left( 0 \right)}{\log R_{1}\left( t \right)-\log R_{1}\left( 0 \right)}$, eqn S3

became

$\frac{a_{clad}}{a_{chir}}=\frac{\log R_{clad}\left( t \right)-\log R_{clad}\left( 0 \right)}{\log R_{chir}\left( t \right)-\log R_{chir}\left( 0 \right)},$ eqn S4

Selectivity can then be assessed by the slope of the trends of the log-transformed prey abundances. To improve comparisons between very steep slopes that tend to be infinitive and very shallow slopes that tend to zero, both representing highly selective predators, we chose to transform $\frac{a_{clad}}{a_{chir}}$ into slope degree angles. Slopes of 45° represent the non-selective opportunistic forager; slopes < 45° represent selective predation of species 1 based on graph a, whereas slopes >45° show selective predation of species 2 (Fig. S1b).


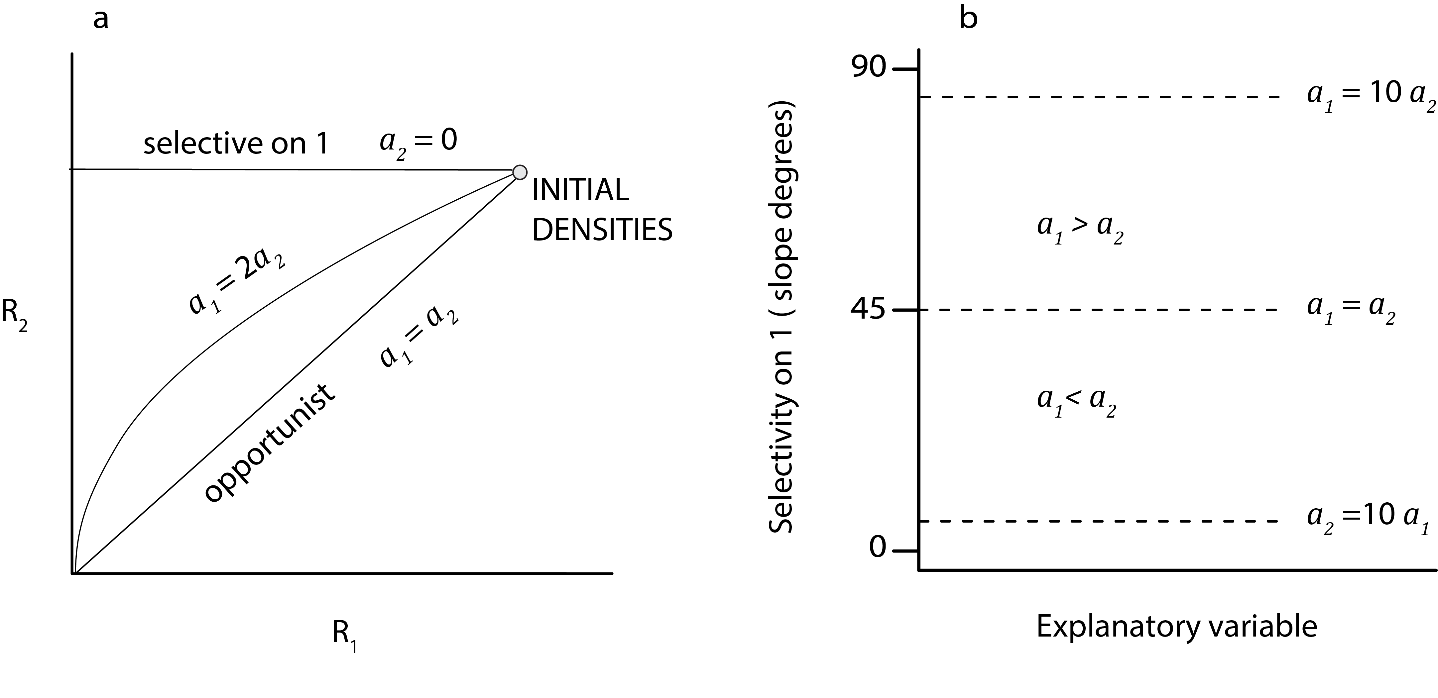


Figure S1. General aspects of our prey selectivity model. a) Graphical representation of prey selectivity (based on relative attack rates *a_i_* on different prey i), given the trajectories of prey-to-prey relationships (redrawn from Holt and Kotler (1987)). R_i_ represent the abundance of prey i. b) Graphical interpretation of our prey selectivity model, which is based on the slopes of joining two consecutive time-points of the log-transformed abundances ($\log R_{i})$ of two prey. Slopes of 45° represent the non-selective opportunistic forager; slopes < 45° represent selective predation of species 1 based on graph a, whereas slopes >45° show selective predation of species 2.

## Estimating Chironomidae depletion

The Chironomidae assemblage was monitored through the emergence of adult stages over time. Therefore, we do not have a direct estimation of Chironomidae abundance in each mesocosm that could be used for estimating selectivity. To solve this, we formulated an empirical model based on the comparison of the total emergence of Chironomidae over the whole experimental period between fish and fishless mesocosms.

We defined the Chironomidae emerging stock $S\left( t \right)$ as the number of emerged Chironomidae that are left from in each mesocosm at a certain time point (equation S5)

$S\left( t \right)=S\left( 0 \right)-P\left( t \right)-E\left( t \right),$ eqn S5

where $S\left( 0 \right)$ is the initial emerging stock in the enclosure, $P\left( t \right)$ is the number of Chironomidae predated between time 0 and time $t$; and $E\left( t \right)$ is the number of Chironomidae that emerged between time 0 and time $t$.

We assumed that the initial Chironomidae emerging stock before fish addition $S\left( 0 \right)$ was the sum of emerging Chironomidae in each of the no fish enclosures such that (equation S6):

$S\left( 0 \right)=\sum_{week=t}^{5} E_{x} (t),$ eqn S6

We also assumed that predation on Chironomidae $P\left( t \right)$ was the difference in emergence for fish and fishless mesocosms for each time point (equation S7)

$P\left( t \right)= E_{x}\left( t \right)-E(t)$, eqn S7

assuming that the phenology of Chironomidae emergence in fish and fishless treatments did not change, at least at the time-scale at which we assessed predator selectivity.

Therefore, the depletion of Chironomidae only due to predation over two consecutive time points was defined as (equation S8):

$\frac{a_{clad}}{a_{chir}}=\frac{\log R_{clad}\left( t \right)-\log R_{clad}\left( 0 \right)}{\log[S\left( 0 \right)-P\left( t \right)]-\log S\left( 0 \right)}$, eqn S8

Representation of the modelled depletion trends for Chironomidae in relation to Cladocera abundance for the different mesocosms can be seen in Fig. S2.

Summary of the assumptions of the model

1. New recruitment of organisms and predation-independent mortality are negligible.
2. Cladocera are predation limited and Chironomidae are predation and emergence limited.
3. In the absence of fish, there are no other predators.
4. $S\left( t \right)$ is a constant proportion of the total Chironomidae assemblage in the system.
5. $S\left( 0 \right)$ is the sum of emerging Chironomidae throughout the experiment in each of the no fish mesocosms, assuming that no fish treatments are true controls of fish mesocosms.
6. $P\left( t \right)$ is the difference in emergence for fish and fishless mesocosms for each time point assuming that the phenology of emergence in fish and fishless treatments does not change.


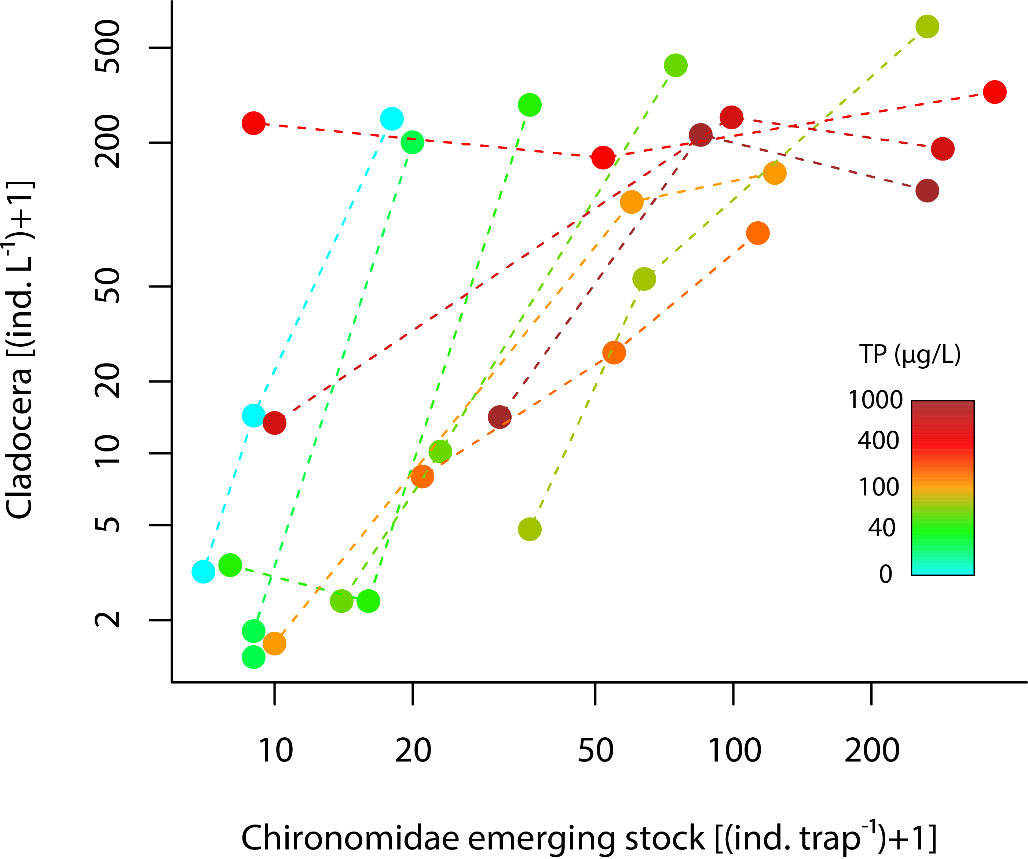


Figure S2. Depletion trends of modelled Chironomidae emerging stock in relation to Cladocera abundance from one week before the predator addition to until 3 weeks after the fish was added. Initial time points for each nutrient level (depicted as total phosphorus, TP; see colour legend) are found on the top-right side of the graph. Subsequent depletion trajectories between points are linked by dashed lines. Note that changes in Chironomidae emerging stock over time are both affected by predation and emergence in this graph, *sensu* Equation S5. However, we only used the effect of predation in Equation S9 to calculate selectivity.

## Hypotheses of predator behaviour

Different predator behaviours will lead to different prey selectivity patterns at changing proportions or absolute abundance of prey (Fig. S3):

- H_0_: the predator acts as an opportunistic forager that would have constant density dependent attack rates for both prey (Figs S1a, S3a).
- H_1_: the predator acts as an optimal forager *sensu* Pyke et al. (1977). Increasing abundance of the preferred prey will lead to a shift from opportunistic foraging to fully selective foraging on the preferred prey (Figs S3b).
- H_2_: the predator acts as a switching forager, increasing density dependent attack rates on the most abundant prey (Fig S3c).
- H_3_: the predator acts as an anti-switching forager *sensu* Abrams (1987), selecting the less abundant limiting prey. This hypothesis fits in a context where predators need to obtain a certain amount of nutrients from one of the prey irrespective to its densities (Fig. 3d).


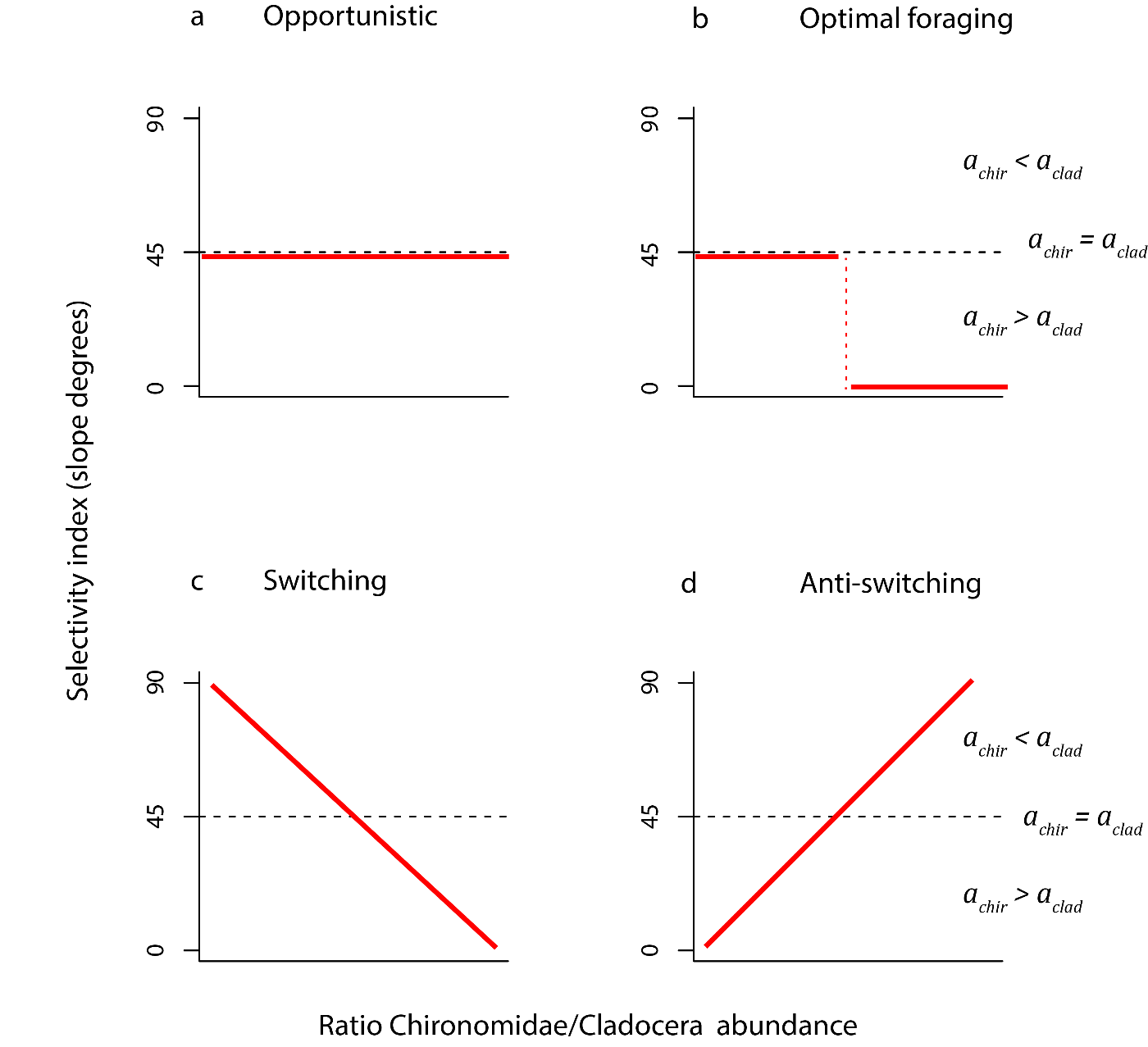


Figure S3. Graphical representations of hypothesis testing different kinds of predator selectivity on Cladocera based on the selectivity index obtained in Equation S9 (red solid lines), compared with the one of non-selective opportunistic predator (dashed black line). A) Hypothesis 0: opportunistic predator throughout the gradient of prey dominance. B) Hypothesis 1: the predator feeds opportunistically until a certain threshold where it feeds solely on the preferred prey (we chose to show Chironomidae as preferred prey in this case). C) Hypothesis 2: switching predator that increasingly selects the prey that increases its dominance in the system. D) Hypothesis 3: anti-switching predator that requires a fixed portion of each prey in their diet. Anti-switching foraging will always cause selective feeding on the least abundant prey.

## Results and interpretation

Selectivity on Cladocera decreased as Chironomidae became more abundant in the community during the first week of the experiment (Fig. S4a-b), (for Chironomidae emerging stock, linear model t = -3.21, p = 0.01; for ratio Chironomidae*/*Cladocera abundance, linear model t = -4.83, p < 0.001). Therefore, these results support H_2_ which stated that the predator follow a switching behaviour (Fig. S2c). These results exclude any other of alternative hypotheses (Fig. S2), since the selectivity index shows no sustained opportunistic behaviour, rejecting H_0_ and H_1_, whereas selectivity toward Cladocera did not increase at decreasing dominance of Cladocera in the system (See Fig. S4b).

In two of the mesocosms at the end of nutrient addition (600 µg L^-1^ total phosphorus and 1000 µg L^-1^ total phosphorus respectively), we found moderately negative slope values (Fig. S4a, b), which arose from higher Cladocera abundances after fish addition. This may have been caused by stochasticity involved in zooplankton sampling and counting, or in small departures from our assumption of no prey recruitment. Still, we did not exclude these points, since similar stochastic events may have happened in previous samples, causing the uncertainty in the linear model.

However, opportunistic foraging did not explain prey selectivity three weeks after fish addition (Fig. S4c, d). On the one hand, this can arise due to increasing stochasticity in the estimations of both Chironomidae and Cladocera abundance when they become less abundant. In addition to this, some of the assumptions of the model may be violated over longer timeframes. For instance, new recruitment of prey communities may increase its influence over prey demographic processes over the long term. Additionally, plastic responses of prey in the presence of the predator usually affect predation susceptibilities, phenology and life-history traits in the presence of fish (e.g. Peckarsky et al., 2002; Merkley et al., 2015), which were assumed to be equivalent in fish and fishless treatments. This might have particularly affected the calculation of $P\left( t \right)$ , which depends on differences in simultaneous emergence in fish and fishless treatments.

Overall, based on this analysis we suggest that crucian carp acted as switching foragers between Cladocera and Chironomidae prey, which agrees on previous knowledge suggesting switching foraging for consumers that couple prey from different habitats (e.g. Begon et al., 2006).


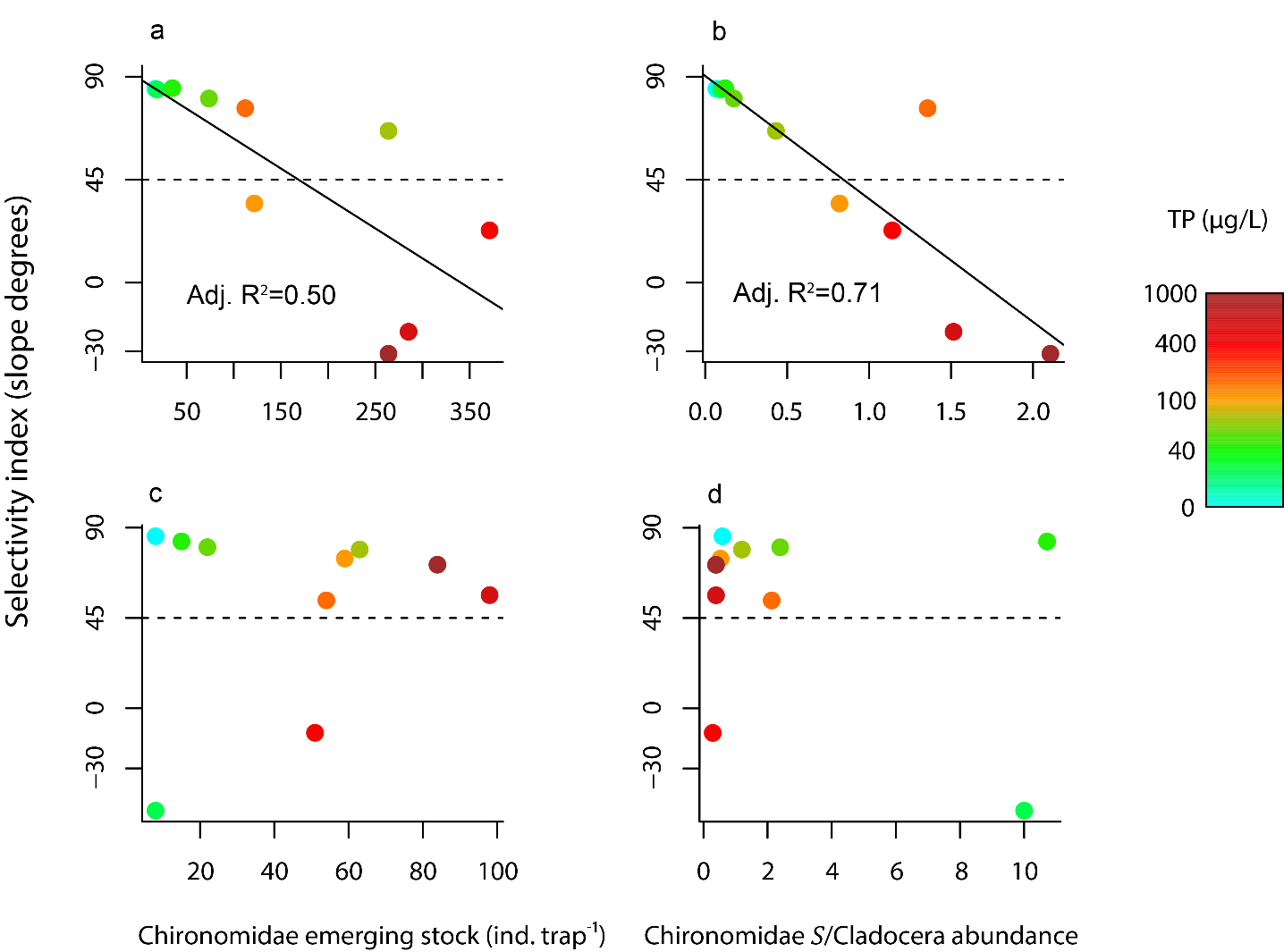


Figure S4. Changes of predator selectivity on Cladocera across changes of absolute (a, c) and relative (c, d) abundance of Chironomidae in the system (as emerging stock, *S*), relative to non-selective opportunistic behaviour (black dashed lines). Graphs a, c represent selectivity on Cladocera based on data from 1 week before and 1 week after the fish addition. Graphs b,d represent selectivity on Cladocera based on data from 1-3 weeks after fish addition. Significant linear relationships of selectivity along the gradients are represented by solid lines, whereby Adj. R^2^ show the adjusted coefficient of determination. The productivity gradient (as total phosphorus, TP) is depicted by different colours in the colour legend.

## References

Abrams, P. A. (1987). The nonlinearity of competitive effects in models of competition for essential resources. *Theoretical Population Biology*, *32*(1), 50–65. https://doi.org/10.1016/0040-5809(87)90039-6

Begon, M., Townsend, C. R., & Harper, J. L. (2006). *Ecology: From individuals to ecosystems* (4th ed). Blackwell Publishing Ltd.

Holling, C. S. (1965). The functional response of predators to prey density and its role in mimicry and population regulation. *The Memoirs of the Entomological Society of Canada*, *97*(S45), 5–60. https://doi.org/10.4039/entm9745fv

Holt, R. D., & Kotler, B. P. (1987). Short-term apparent competition. *The American Naturalist*, *130*(3), 412–430. https://doi.org/10.1086/284718

Merkley, S. S., Rader, R. B., & Schaalje, G. B. (2015). Introduced Western Mosquitofish (Gambusia affinis) reduce the emergence of aquatic insects in a desert spring. *Freshwater Science*, *34*(2), 564–573. https://doi.org/10.1086/680381

Peckarsky, B. L., McIntosh, A. R., Taylor, B. W., & Dahl, J. (2002). Predator chemicals induce changes in mayfly life history traits: A whole-stream manipulation. *Ecology*, *83*(3), 612–618. https://doi.org/10.1890/0012-9658(2002)083[0612:PCICIM]2.0.CO;2

Pyke, G. H., Pulliam, H. R., & Charnov, E. L. (1977). Optimal foraging: A selective review of theory and tests. *Quarterly Review of Biology*, *52*(2), 137–154. https://doi.org/10.1086/409852
